# Phenotypic Heterogeneity of *Pseudomonas aeruginosa* Populations in a Cystic Fibrosis Patient

**DOI:** 10.1371/journal.pone.0060225

**Published:** 2013-04-03

**Authors:** Matthew L. Workentine, Christopher D. Sibley, Bryan Glezerson, Swathi Purighalla, Jens C. Norgaard-Gron, Michael D. Parkins, Harvey R. Rabin, Michael G. Surette

**Affiliations:** 1 Farncombe Family Digestive Health Research Institute, McMaster University, Hamilton, Ontario, Canada; 2 Department of Medicine, McMaster University, Hamilton, Ontario, Canada; 3 Department of Biochemistry and Biomedical Sciences, McMaster University, Hamilton, Ontario, Canada; 4 Department of Microbiology, Immunology and Infectious Diseases, University of Calgary, Calgary, Alberta, Canada; 5 Department of Medicine, University of Calgary, Calgary, Alberta, Canada; 6 Adult Cystic Fibrosis Clinic, University of Calgary, Calgary, Alberta, Canada; Louisiana State University and A & M College, United States of America

## Abstract

The opportunistic pathogen *Pseudomonas aeruginosa* chronically infects the lower airways of patients with cystic fibrosis. Throughout the course of infection this organism undergoes adaptations that contribute to its long-term persistence in the airways. While *P. aeruginosa* diversity has been documented, it is less clear to what extent within-patient diversity contributes to the overall population structure as most studies have been limited to the analysis of only a few isolates per patient per time point. To examine *P. aeruginosa* population structure in more detail we collected multiple isolates from individual sputum samples of a patient chronically colonized with *P. aeruginosa*. This strain collection, comprised of 169 clonal isolates and representing three pulmonary exacerbations as well as clinically stable periods, was assayed for a wide selection of phenotypes. These phenotypes included colony morphology, motility, quorum sensing, protease activity, auxotrophy, siderophore levels, antibiotic resistance, and growth profiles. Each phenotype displayed significant variation even within isolates of the same colony morphotype from the same sample. Isolates demonstrated a large degree of individuality across phenotypes, despite being part of a single clonal lineage, suggesting that the *P. aeruginosa* population in the cystic fibrosis airways is being significantly under-sampled.

## Introduction

Cystic fibrosis (CF) is an autosomal recessive disorder where mutations in the CF transmembrane conductance regulator lead to altered ion flux and the reduction of fluid covering the surface of airway epithelial cells [1]–[3]. Compromised innate immunity including reduced mucociliary clearance in patients with CF, gives a variety of species the opportunity to colonize the lower airways, ultimately generating long-term complex bacterial infections [2], [4]–[6]. The microbial community in the CF lung is known to be complex[7]–[15] and its changing dynamics can influence severity of disease [10], [16]. However, *Pseudomonas aeruginosa* infection is traditionally considered to be the most common agent contributing to lung function decline and ultimate mortality in CF patients [17]–[19]. *P. aeruginosa* infections in the lower airways can be maintained for decades. Throughout this time *P. aeruginosa* exhibits genetic and phenotypic diversity thought to occur as the organism adapts to the lower airway environment [20], [21]. Some factors considered to be the hallmarks of *P. aeruginosa* adaption to the CF airways include a switch to the mucoid phenotype [22] and loss of motility [23]. Diverse colony morphologies from clonal populations of *P. aeruginosa* are also routinely observed in clinical laboratories and some have been linked to increased antibiotic resistance and enhanced cytotoxicity [24]–[26]. It has been suggested that these colony variants have adaptations specific for persistence in the lower airways [27].

Although such genetic and phenotypic changes have been documented, cross-sectional studies are often limited to a small number time points from a large number of patients [28] and longitudinal studies by a large number of time points from a small number of patients [20] thus the full extent of micro-evolutionary dynamics that might be present throughout the course of infection is not yet well defined. Furthermore, most studies have analyzed a limited number of isolates per sample. Recent evidence suggests that this leads to an underestimation of the true diversity present in a sample. For example, Wilder and colleagues examined quorum-sensing phenotypes in clinical *P. aeruginosa* isolates and found very high within-patient diversity [29]. In addition, large variation in antibiotic resistance profiles was found for *P. aeruginosa* isolates with the same morphotype [30], [31]. By way of screening multiple phenotypes Mowat *et al*. have demonstrated high intraspecific diversity in the *P. aeruginosa* Liverpool Epidemic Strain (LES) that is responsible for a significant burden of infection in both Europe and North America [32]. One implication of high intraspecific diversity in the CF airways is that analysis of only a few isolates may lead to a misrepresentation of the *P. aeruginosa* phenotype, such as oversimplified antibiotic resistance profiles. Therefore a clear understanding of *P. aeruginosa* evolutionary dynamics in the CF airways that includes intraspecific diversity is necessary.

In the present study we examined the population dynamics of a newly described transmissible strain of *P. aeruginosa* [33] in a single patient over the course of one year. A number of phenotypes linked to survival and persistence in the CF airways were measured including colony morphology, motility, quorum sensing, proteases, auxotrophy, siderophores, growth profiles and antibiotic resistance. This represents more phenotypes than have been previously examined in single studies of *P. aeruginosa* intraspecific diversity. We observed very high diversity across all phenotypes even within isolates of a single colony morphotype recovered from a single sputum sample, which has been noted previously in LES epidemic *P. aeruginosa* strain [32]. However, we find that each phenotype is highly variable between isolates and not accurately characterized as simply the presence or absence of a phenotype. No obvious patterns in phenotype prevalence or activity was strongly correlated with early exacerbation samples or periods of clinical stability. This suggests that *P. aeruginosa* contribution to acute exacerbation may arise in part through complex population interactions rather than fluctuations in specific virulent strains within the population.

## Results

**Figure 1:**
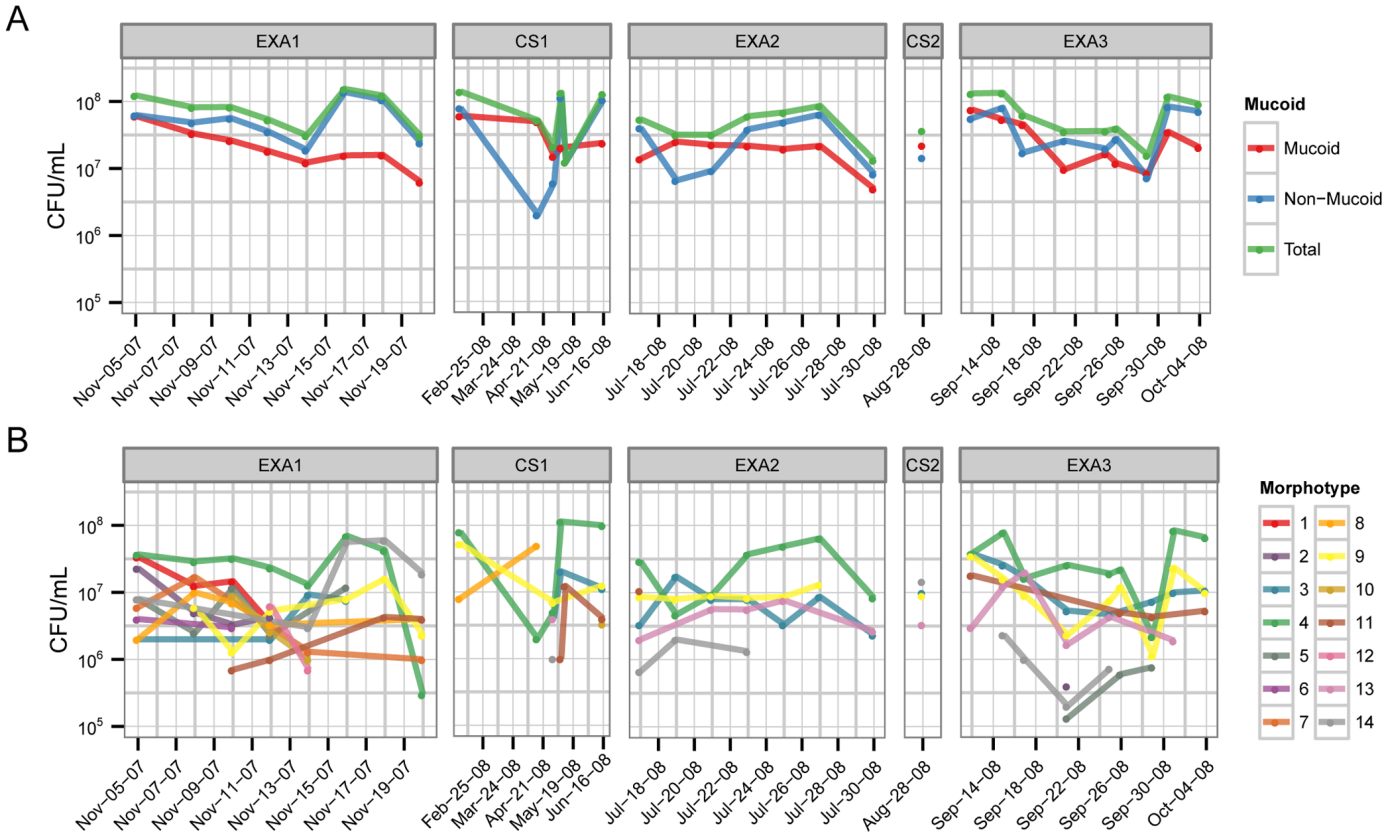
Levels of *P. aeruginosa* during three pulmonary exacerbation periods over the course of one year. A, Total levels, expressed as colony forming units (CFU) per mL of sputum are shown in green and the numbers of mucoid and non-mucoid isolates are indicated in red and blue, respectively. B, The CFU per ml for each of the 14 colony types are shown.

The source patient was a 31 year old male (genotype F508del/F508del) with advanced CF airways disease, whom had been chronically infected with *P. aeruginosa* for 20 years. Thirty-four expectorated sputum samples were collected from the patient over one year, and comprised 16 samples collected during periods of clinical stability and 18 samples collected through three acute pulmonary exacerbations during which the patient was hospitalized and treated with parenteral anti-pseudomonal therapies (Table S1). Multiple *P. aeruginosa* isolates were collected from each sample as well as multiple isolates with identical colony morphologies (representative plate image shown in Figure S1). To ensure that slow growing colonies were not overlooked the plates were allowed to incubate for 72 h, which is longer than usual for a clinical lab. Fourteen unique colony morphologies were identified in a total of 169 isolates and were comprised of equal numbers of mucoid and non-mucoid isolates (Figure 1A). The cell counts for each colony type varied greatly throughout the exacerbation periods and no associations between exacerbation and a particular colony type were observed (Figure 1B). We also noted for all three exacerbations that the total levels of *P. aeruginosa* did not show any decrease, despite antibiotic therapy and resolution of symptoms.

**Figure 2:**
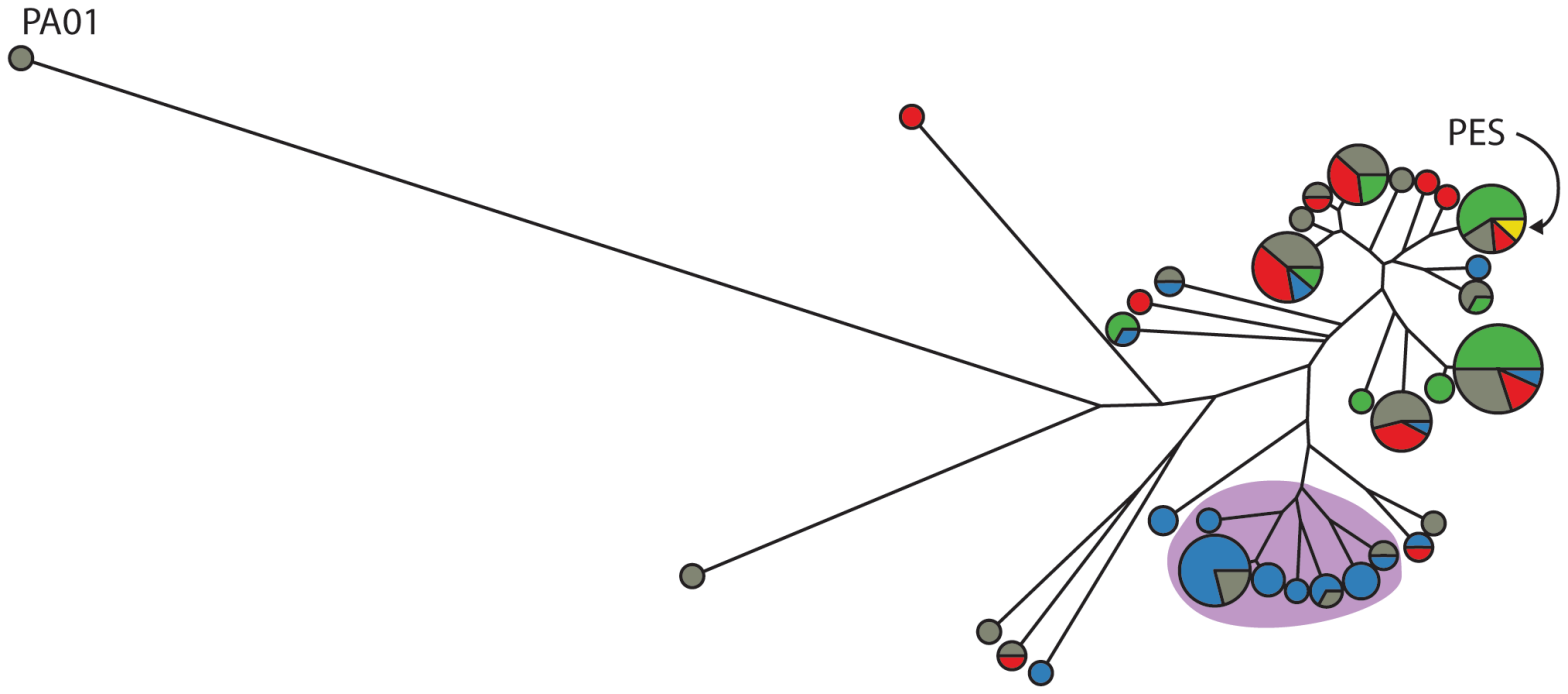
Genetic diversity present in collected *P. aeruginosa* isolates from a single patient. The Dice coefficient from pulse field gel electrophoresis patterns of each isolate was used to construct a UPGMA tree (unrooted). Isolates from this study were no more than 20% different from each other but were more than 40% different from PA01. Isolates with identical PFGE patterns are grouped into a single node where the size of that node represents the number of isolates. Colouring represents the phenotypic group that the isolate falls into and the colours match the colouring in Figure 5. Two PES strains were included in the analysis and are coloured in yellow. The cluster highlighted by purple is a cluster where the phenotypic and genetic groups are similar.

The genetic variability of this population was monitored using pulse field gel electrophoresis (PFGE). The pulse-field patterns reveal that all isolates belong to a single clonal group with only 2–3 band differences between them (Figure 2 and Figure S2), which is indicated by a Dice coefficient of greater than 80% for all the pairwise combinations of isolates. The PFGE patterns of the isolates matched the pattern of a recently identified epidemic strain (highlighted in yellow in Figure 2 and indicated in Figure S2), the Prairie Epidemic Strain (PES), which is known to be present in ∼30% of the patients in the clinic that the source patient was attending [33].

**Figure 3:**
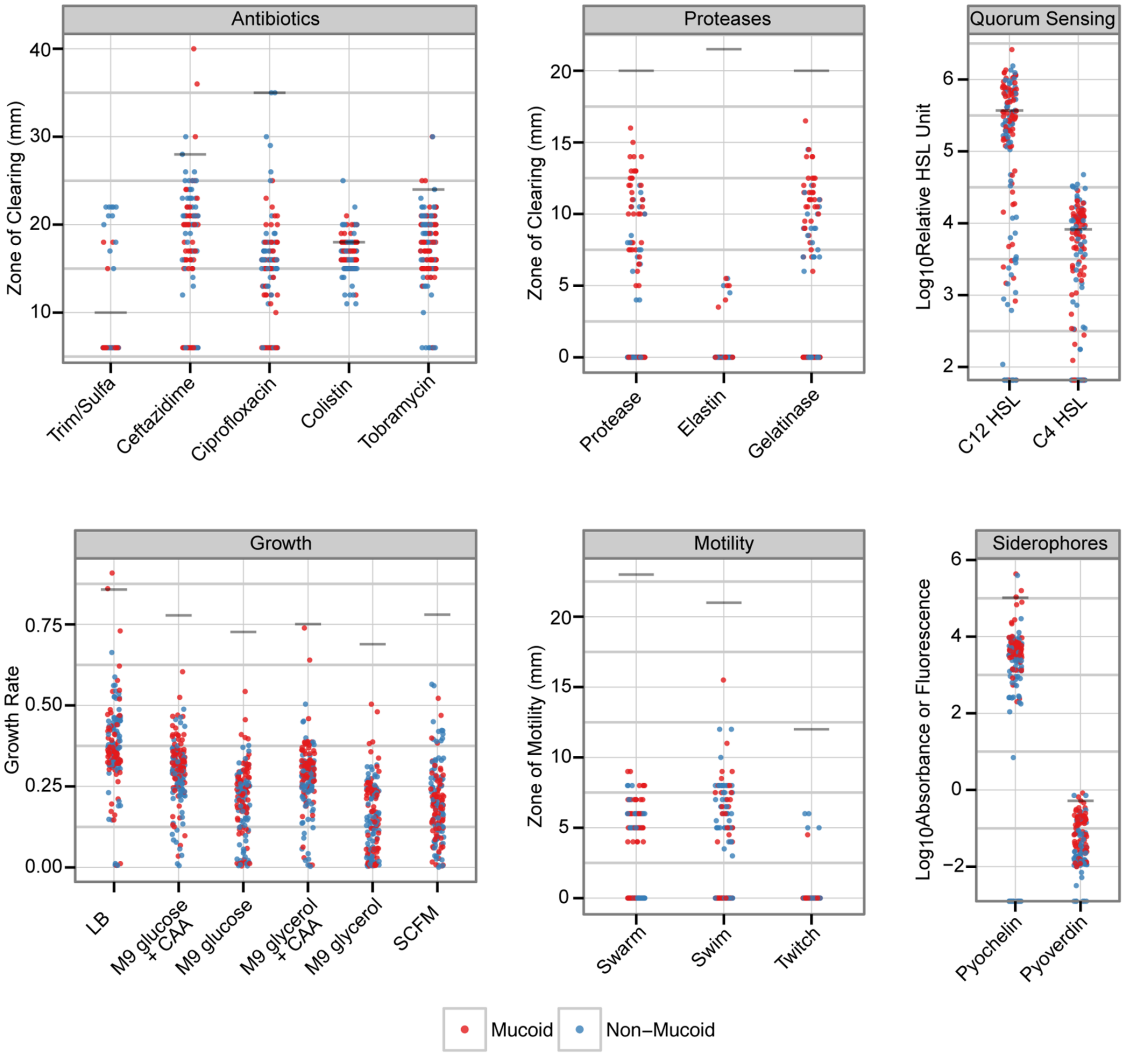
Phenotypic diversity present in collected *P. aeruginosa* isolates from a single patient. The data points are coloured according to whether the isolate was classified as mucoid or not. Grey lines indicate the phenotype value measured for the common lab strain, PA01.

To assemble phenotype profiles we measured 21 different phenotypes related to virulence, including swimming, swarming and twitching motility, activity of several proteases, quorum sensing signal molecule production, siderophore levels, antibiotic resistance to five antibiotics, and growth profiles in different media types, including nutrient rich medium, minimal medium, synthetic CF medium and medium lacking amino acids to measure the prevalence of auxotrophy. The most prominent feature that emerged from this analysis (summarized in Figure 3) is the large amount of variance within each phenotype. Each isolate demonstrated a high degree of individuality from the other isolates (i.e. no two isolates were the same) and we observed a large range in values for every phenotype measured beyond the normal variation inherent to the assays (Figure S3). We also noted that although some phenotypes, such as gelatinase and protease activity were highly correlated, for the most part, phenotypes behaved independently (Figure S4). Moreover, there were no significant changes in mean phenotype values over time (Figure S5). It is also interesting to note that there were approximately equal numbers of motile and non-motile organisms. In the swimming assay 51% of all the isolates were motile and in the swarming assay 56% were motile. There was also an approximately equal number of mucoid and non-mucoid colony types in both the motile and non-motile isolates (data not shown). That is to say, loss of motility was not correlated to mucoidy.

**Figure 4:**
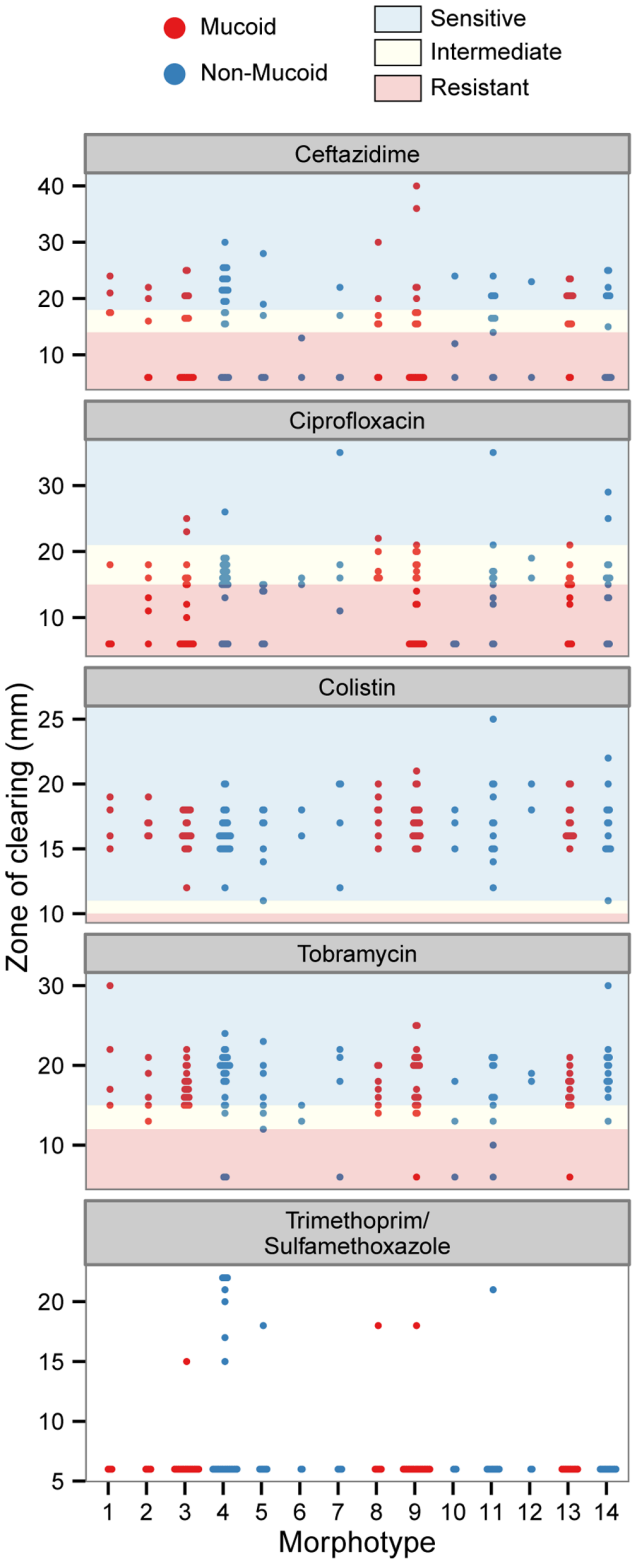
Distribution of antibiotic resistance among the analyzed isolates. Shown are zones of clearing from disc diffusion assays for five commonly used antibiotics in CF clinics and the resistance cutoffs for each antibiotic are given by the dashed lines. Resistance profiles for each colony morphotype are shown individually and coloured according to whether that colony type was initially identified as mucoid or not. Cutoff values for resistant, intermediate and sensitive are indicated by the colour of the background; red, yellow and blue, respectively. Cutoff values for trimethoprim/sulfamethoxazole are not published for *P. aeruginosa* by the CLSI.

Disk diffusion assays were used to assess antibiotic susceptibility, which also showed a high level of variation (Figure 4). Many of the morphotypes had both sensitive and resistant isolates but even among the sensitive isolates there was large variability in the zone of clearing (colistin is a good example of this). Although all isolates were susceptible to colistin there was a range of zone sizes observed, with some approaching resistant. Importantly, this patient was exposed to colistin for only a few weeks during a clinically stable period and had no colistin exposure during exacerbation periods that samples were collected from (Table S1). In this set of isolates there was no association with acute antibiotic exposure during exacerbation and the abundance of resistant isolates in the population (Figure S6). We also noted that neither morphotype nor mucoidy were associated with any particular antibiotic resistance profile (Figure 4).

**Figure 5:**
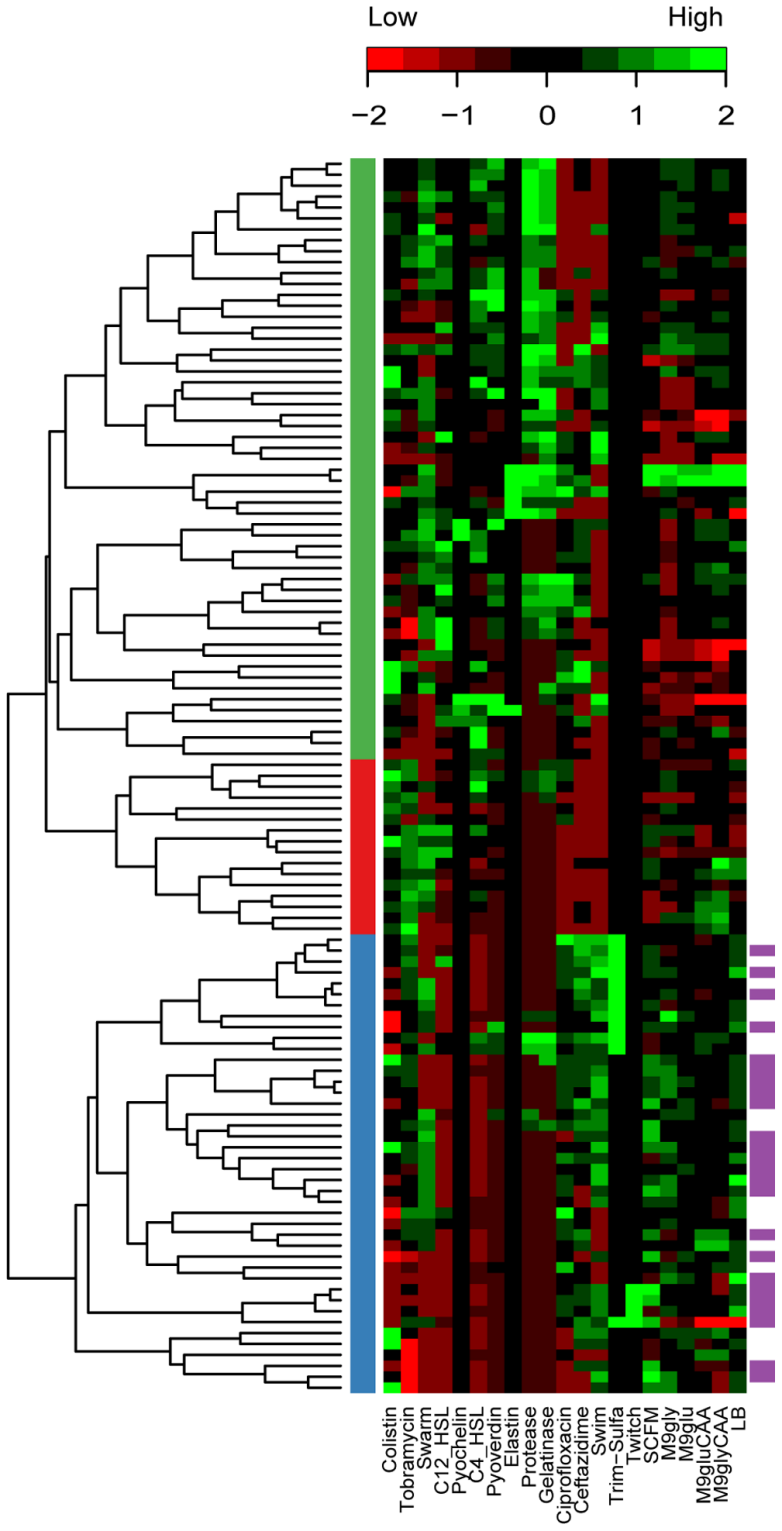
Hierarchical cluster analysis of the phenotype profiles. Green indicates a value above the mean and red indicates a value below the mean. The three colours on the left side indicate the three main cluster groups. Purple bars on the right side indicate the isolates that group closely in both the phenotypic and genetic analysis. Missing values were removed prior to analysis.

A clustering analysis of all the phenotypes measured (Figure 5) reveals a high level of individuality present in these isolates. What is noteworthy from the clustering is that the isolates do not cluster by morphotype or mucoidy or any of the other metadata variables, including date of isolation (not shown). Rather, the isolates clustered into 3 main phenotypic groups, which can also be seen in the principle components analysis (PCA) (Figure S7). To determine if there was any correlation between the genetic (PFGE profile) and phenotypic measures each isolate in the UPGMA tree was coloured according to the phenotypic groups it fell into (Figure 2). In general there was not good agreement between the genetic and phenotypic clustering with the exception of one group of isolates. The group is highlighted in purple in both the genetic (Figure 2) and phenotypic (Figure 5) data. The functional importance of any groups remains to be explored, however, these findings do highlight the depth of diversity even within a single morphotype.

**Figure 6:**
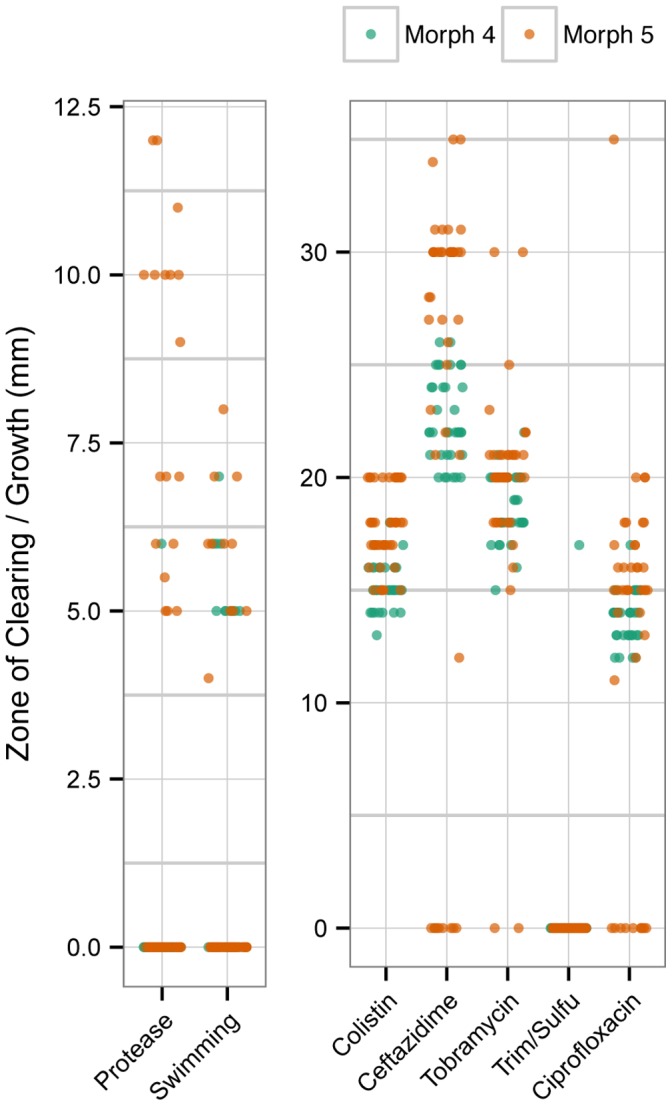
Phenotypic diversity of *P. aeruginosa* isolates from a single sample. Phenotype profiles were obtained from a total of 88 isolates and were comprised of two different colony morphologies, which are shown as orange and green on the plot.

The diversity present within a single sample was analyzed more in depth by selecting 44 individual isolates from each of two different morphotypes (4 and 5) from one sputum sample that was collected at later date. For these additional 88 isolates we measured a subset of phenotypes; antibiotic sensitivity, protease production and swimming motility. As shown in Figure 6 there was a large degree of phenotypic variation even within isolates from a single sample and of a single colony morphotype. Both morphotypes had motile and non-motile isolates and morphotype 5 had both protease positive and negative isolates. Both morphotypes also had a large distribution of antibiotic sensitive isolates and with the exception of colistin, a good number of resistant isolates. The large phenotypic variation observed in similar morphotypes was still present at a higher sampling depth, indicating that even with this number of isolates under-sampling is still occurring.

To verify that such high diversity is not limited to a single patient, samples were collected from an additional 3 patients during a single pulmonary exacerbation. We found high phenotypic diversity among the *P. aeruginosa* isolates from all patients examined, which were comprised of both PES and non-PES strains (Figure S8). Levels of *P. aeruginosa* were monitored throughout the course of the exacerbation and we found that in one of the three patients *P. aeruginosa* levels did not decrease (Figure S9C), as we observed for the main patient (Figure 1A and Figure S9D). However, in two of the patients there was a noticeable drop in *P. aeruginosa* levels near the end of the exacerbation period (Figure S9 A and B). For all patients examined we found that levels of mucoid and non-mucoid isolates were approximately the same and this did not change during exacerbation periods (Figure S9). Furthermore in the patients where the levels of *P. aeruginosa* dropped during the exacerbation we did not observe any corresponding decrease in measured antibiotic resistance (data not shown), however, deeper sampling will be required to fully resolve this.

## Discussion

The phenotypic variability in *P. aeruginosa* isolates from chronic CF patients is documented in the literature. *P. aeruginosa* isolates, even clonal isolates from the same sample, have been shown to have large variations in motility [34], [35], quorum sensing [29], [34], virulence factor production [34]–[36], antibiotic susceptibility [30], [31], [36] and biofilm formation [34]. A recent study examined phenotypic and genetic variability in the *P. aeruginosa* Liverpool Epidemic Strain (LES) from 10 adult CF patients and found large diversity in virulence factors, auxotrophy, hypermutability, antibiotic resistance, presence of prophage, and colony morphotype [32]. Here we expand the range of phenotypes examined and identify high phenotypic diversity within a novel epidemic strain, PES. There are several key similarities between the LES and PES isolate collections. First, both demonstrate a high degree of variability within isolates from a single sample, indicating the need for deeper sampling. In addition, the heterogeneity in antibiotic resistant profiles of both sets of isolates remained consistent during and after antibiotic treatment, indicating a lack of positive selection for resistance or negative selection for sensitivity. An important observation from our analysis of the PES isolates is that binning a phenotype into categories such as ‘high’ or ‘low’ will lead to the underestimation of the true diversity in a sample. For example even though many of our isolates would be classified as sensitive to a number of antibiotics there was still variability within the measured zones of clearing. As such, if isolates are to be clustered for further analysis, as done by Mowat *et al.,* [32], binning the phenotypes will underestimate the diversity and may not represent an accurate population structure. What is clear however, from the current study and others [29], [30], [32], [36] is that the *P. aeruginosa* population is being under-sampled based on morphotype assessment alone. How many isolates per sample are required to accurately describe the population structure and how best to measure diversity are significant challenges.

Many studies have also documented the genetic variability inherent to *P. aeruginosa* during chronic lower airway infections. Mutational targets often identified are *mexZ*, *lasR,* and *mucA*, a regulator of alginate biosynthesis [20], [28], [37]–[39]. For some time many *P. aeruginosa* adaptations have been associated with adaptation to and chronic persistence of the bacterium in the lower airways. Examples of this include loss of motility, thought to be associated with decreased immunogenicity [23], [40], and conversion to the mucoid phenotype, a key indicator of poor prognosis [22], [41], [42]. Evidence presented in the current study suggests that non-mucoid isolates are just as prevalent as mucoid isolates. Similarly, we found both motile and non-motile isolates in relatively equal abundance, which has been observed previously [32], [35], [36]. The implication of this is that rather than a single phenotype dominating the lung environment, a number of phenotypes can evolve, including but not restricted to, mucoid phenotypes. Given that environmental heterogeneity is very good explanation for the maintenance of diversity [43], the rapid emergence of large diversity in the *Pseudomonas* population is suggestive of many diverse niches within the lower airways leading to a number of sub-populations [29]. It is also important to note that expectorated sputum by its very nature is not necessarily representative of the entire lung and we do not know whether it represents a sample from one lobe or a mixture (and will vary between samples). However, most of our samples were collected during physiotherapy and therefore may be more representative than single spontaneous sputum samples.

Adaptive radiation, the evolution of multiple lineages to fill available niches [44], [45], is perhaps the most attractive theory to account for the emergence of such diversity in lower airway *Pseudomonas* populations. Lee *et al.,* observed large diversification of *E. coli* populations in monoassociated mice over a period of 1000 days, which corresponded to approximately 20,000 *E. coli* generations [46]. In a very reproducible manner, *P. fluorescens* rapidly diversifies into multiple niche specialists within a matter of days [47]. In *Salmonella*, laboratory passage leads to rapid loss of a spatial phenotype and accumulation of *rpoS* mutations [48]. The evolved populations exhibited a complex distribution of *rpoS* activity indicating that many different types of mutations were being generated even in the absence of spatial heterogeneity [48]. These examples highlight how spatial heterogeneity and niche partitioning can drive diversification, both of which are relevant in the complex and dynamic CF airways. It is important to note as well, that it is not clear if underlying cause of the phenotypic diversity is exclusively genetic in origin or partly a result of phenotypic plasticity. Although there were certainly genetic differences between the isolates, PFGE was not able to distinguish all the individual isolates due to inherent lack of resolution.

In conclusion, the high intraspecific diversity of *P. aeruginosa* populations demonstrated here and in other studies, indicates that caution is in order when making conclusions based on the analysis of a small number of samples. Assuming that one (or a few) isolates is representative of the entire population can lead to underestimations of the true diversity present. This may also lead to inaccurate descriptions of *P. aeruginosa* phenotypes such as antibiotic resistance, which would have significant implications for antibiotic therapy [31]. There are also implications for detailed genetic and physiological studies that usually focus on a single isolate [27]. Such studies can provide useful information, however, to extend these conclusions to the whole population may not be accurate. Furthermore, from an ecological standpoint, underestimating the diversity can also hinder attempts to fully understand the population structure and persistence of this organism in the lower airways. Given the polymicrobial nature of the CF lower airway infection it will be paramount to understand how *P. aeruginosa* diversity can influence the population structure of the whole polymicrobial community and vice versa. *P. aeruginosa* contribution to disease progression in CF may require an understanding of the interaction of a complex population of *P. aeruginosa* as a whole with the host and other microbes. Variants within the *P. aeruginosa* population may contribute to different aspects of disease progression such as the transition to a pulmonary exacerbation. Furthermore we know that other organisms in the lower airway community can modulate *P. aeruginosa* virulence [49], [50] and it may be that the key to understanding these infections lies in the interplay between the various community members, including diverse *P. aeruginosa* sub-populations.

## Materials and Methods

### Ethics Statement

The research performed in this study including sample collection and subject consent procedures has been approved by the Conjoint Health Research Ethics Board of the Faculties of Medicine, Nursing and Kinesiology, University of Calgary (CHREB), approval number E-23087: Collection, Storage and Banking of Tissue Samples (Blood and Sputum) and Sputum Microbial Isolates of Patients Attending the Adult Cystic Fibrosis Clinic, University of Calgary Medical Clinic of the Foothills Medical Center for the purposes of CF research. Subject consent was provided in written format through an informed consent form approved by the CHREB.

### Sample collection and bacterial isolation

Starting in the fall of 2007 serial sputum samples were collected from an adult CF patient from the Southern Alberta Adult CF clinic. After physical therapy targeting the four quadrants of the lungs, sputum was ptyalized four times and pooled into sterile containers. Within a period of two hours after collection, sputum samples were sheared by passage through a 1-cc needleless syringe to help emulsify the sample and then serially diluted in nutrient rich brain heart infusion (BHI) broth (BD). The 10^−3^, 10^−4^ and 10^−5^ dilutions were first rigorously vortexed and 300 µL was plated on large (150 mm diameter, VWR) MacConkey (BD) (MAC) agar plates. After incubation for a minimum of 72 h and up to 96 h at 37°C, plates were visually inspected for growth of *P. aeruginosa* and morphotypes were identified based on colony size, pigment production, texture and mucoidy. A representative colony of each morphotype was purified three times on MAC plates and on Pseudomonas Isolation Agar (BD) (PIA) to confirm that the isolated colony was *P. aeruginosa*. Upon purification, colony morphology was compared to the morphology found on the initial sample plate. Morphotype stability of each isolate was checked by plating isolates on MAC plates and inspecting for changes in colony morphology after 48 hours of incubation at 37°C. Isolates were frozen at −80°C in 10% (w/v) skim milk powder (BD). One hundred sixty-nine *P. aeruginosa* isolates were collected through three exacerbations and 7 separate samples collected from routine visits to the CF clinic. An additional 88 isolates, comprised from two colony types, were collected at a later date from a single sputum sample.

For storage and routine assays the isolates were arrayed in multi-well plates containing TSB broth with 20% (w/v) glycerol and frozen at −80°C. For phenotyping, multi-well plates containing TSB or BHI were inoculated from the frozen storage plates using a pin replicator. Plates were then sealed with sealing tape and incubated for 24 or 48 hours with shaking at 37°C. After incubation, cultures were diluted (1/10) into 100 µl of fresh media and incubated for a further 4 hours at 37°C before being used as inoculants for phenotype assays. All isolates exhibited reproducible phenotypes when assayed as described. Many of the assays (e.g. motility) show day-to-day variability due to subtle differences in conditions but the relative phenotype measurements compared to control strains were consistent.

### Phenotype Assays

Phenotype assays were performed according standard protocols previously described in the literature and summarized below. Full method details can be found in the supplementary information. Protease production was measured using dialyzed brain heart infusion (D-BHI) milk media [51]. Gelatinase and elastin activity were assayed using previously described assays [52], [53]. Swimming motility of *P. aeruginosa* was investigated by using 0.3% (w/v) agar LB plates and swarming was assayed using nutrient glucose media solidified with 0.5% (w/v) agar [54]. Twitching was measured using thin LB media without salt (LBNS) [55]. *P. aeruginosa* was tested for resistance to commonly used anti-pseudomomal drugs using a modified Kirby Bauer disk diffusion method. Five common anti-pseudomonal antibiotics at clinically relevant concentrations were tested, ceftazidime 30 µg (CAZ, Sigma, Aldrich); ciprofloxacin 5 µg (CIP, Sigma, Aldrich), tobramycin 10 µg (NN, Sigma, Aldrich), trimethoprim/sulfamethoxazole 1.25 µg/23.75 µg (SXT, Sigma, Aldrich), colistin 10 µg (CL, Sigma, Aldrich). Zone diameters of clearing were measured and isolates were classified as described by the Clinical Laboratory Standards Institute, (formerly National Committee on Clinical Laboratory Standards) guidelines as resistant, intermediate or susceptible to the antibiotic [56]. Both 3-oxo-C12 HSL and C4 HSL autoinducer production of *P. aeruginosa* isolates were measured using previously described assays [57]. Growth in auxotrophic conditions was tested with M9 minimal media supplemented with either 0.5% (w/v) dextrose or glycerol, with and without 0.2% (w/v) casamino acids (CAA). Growth in high nutrient media was measured using LB media and growth was also measured in synthetic CF medium [58]. Siderophores were directly measured from spent media of cultures that were grown in M9 minimal media supplemented with glucose and CAA as described [59]. The presence of pyoverdin was assayed by measuring absorbance at 405 nm and pyochelin was assayed by measuring fluorescence with an excitation of 355 nm and emission at 460 nm. Raw data for the phenotype measurements is available upon request.

### Data analysis

For comparison across different phenotypes mean measurements were normalized by unit variance scaling (dividing each value by the standard deviation of that phenotype) and then by mean centering. Principal component analysis and clustering using the Pearson correlation and un-weighted pair group average (UPGMA) was done in R [60]. Growth curves were fit using a spline method and growth parameters estimated from the fits [61]. Maximum growth rates were used for further analysis.

### Pulse-field gel electrophoresis

Strains were inoculated from frozen stocks onto BHI agar and cultured at 37°C for 48 hours. A cell suspension equivalent to a 1.5 MacFarland standard was prepared in suspension buffer (1 M NaCl, 10 mM Tris-HCL, pH 7.6) and mixed with an equal volume of molton 1% (w/v) SeaKem Gold Agarose in TE_1_ buffer (10 mM Tris-HCL, 1 mM EDTA, pH 7.6). This mixture was added immediately to a prepared PFGE plug mold (BioRad). Once the plugs were solidified the embedded cells were lysed for 4 h at 37°C in lysis solution (1 M NaCl, 100 mM EDTA, 0.5% Brij-58, 0.5% sarcosyl, 0.2% deoxycholate, 6 mM Tris-HCl [pH 7.6], 1 mg/mL lysozyme, 20 µg/mL RNase). This was followed by an overnight incubation at 50°C in ESP solution (0.5 M EDTA [pH 9], 1% sarcosyl, 70 µg/mL proteinase K) to digest proteins. Lysed plugs were washed 5 times in TE_0.1_ (T10 mM Tris-HCL, 0.1 mM EDTA, pH 7.6) to remove all traces of lysis and ESP solutions. Plugs were stored temporarily at 4°C in TE_0.1_. Prior to restriction digest the plugs were incubated with 1X reaction buffer (NEBuffer 4) for 15 min at room temperature to allow the buffer to fully penetrate the plug. Each plug was digested with 20U *SpeI* (New England Biolabs) for 4 h at 37°C in 1X reaction buffer with agitation every hour to ensure complete restriction.

Electrophoresis was performed using the BioRad CHEF Mapper PFGE system according to manufacturers instructions. 1% agarorse gels were prepared with SeaKem Gold agarose in 0.5X TBE. Plugs were loaded along with the lambda ladder (New England Biolabs) and overlayed with more agarose. Running conditions were as follows: running buffer: 0.5X TBE, temperature: 10°C, initial switch time: 5 sec, final switch time: 45 sec, included angle: 120°, cell voltage: 6 V/cm, run time: 20 hours. Gels were stained for 60 min with 0.5X GelRed fluorescent nucleic acid stain (Biotium), rinsed 3X in distilled water and imaged on a UV transilluminator.

Gel images were inverted and the contrast and brightness optimized in Adobe Photoshop using the ‘Curves’ tool. Optimized images were analyzed with BioNumerics (Applied Maths). After gel normalization bands were assigned manually. Sample lanes were compared using the Dice coefficient, allowing for a 1% tolerance in band matching. Hierarchical clustering was performed using the un-weighted pair group average (UPGMA) linkage and selecting the highest scoring tree from 200 permutations.

## Supporting Information

Figure S1 **Representative plate showing the diversity of colony morphologies of** ***P. aeruginosa*** **isolates from a single sputum sample.**
(PDF)

Figure S2 **Pulse-field gel electrophoresis.** A UPGMA tree based on PFGE patterns across all the isolates compared using the Dice coefficient. The tree is identical to the one shown in Figure 2, but has been rooted to allow for display of the gel images.
(PDF)

Figure S3 **Variation in growth measures.** Growth rates for each isolate under different media types are shown with error bars (standard deviation). Isolates were sorted according to their growth in M9 minimal media with glucose and case amino acids.
(PDF)

Figure S4 **Correlation matrix of the measured phenotypes.** The Pearson correlation coefficient was calculated for each phenotype pair where the yellow represents a high correlation (close to 1) and blue represents no correlation (close to 0).
(PDF)

Figure S5 **Changes in mean phenotype values over time.** The mean of each phenotype was calculated for each sputum sample. The point are coloured according to the clinical status. A local smoothing function, shown with a blue line (shaded area represents 95% confidence intervals) was added to identify any trends present in the data.
(PDF)

Figure S6 **Antibiotic susceptibility profiles of the collected isolates during the three pulmonary exacerbations.** Isolates were classified based on zone sizes from disk diffusion assays. For each sputum sample the relative proportion of resistant (red), intermediate (blue), and sensitive (green) isolates is shown for the four antibiotics where standardized cutoff values for *P. aeruginosa* are available.
(PDF)

Figure S7 **PCA analysis of the measured phenotype profiles.** The first two components are shown and the data points are coloured according to the cluster pattern observed in the hierarchical cluster analysis. The amount of variation (in percent) explained by each principle component is shown on the axis.
(PDF)

Figure S8 **Phenotypic diversity of** ***P. aeruginosa*** **isolates from multiple CF patients.** Isolates were collected from an additional 3 CF patients to determine the extent of phenotypic diversity. Samples were collected during a single exacerbation period and processed as before. Multiple isolates were collected from each sample, which often contained both mucoid (red) and non-mucoid (blue) isolates. Patient 1 was colonized with a non-PES strain while both patients 2 and 3 were colonized with PES.
(PDF)

Figure S9 **Responders versus non-responders.** Cell counts for mucoid (red), non-mucoid (blue), and total (green) *P. aeruginosa* in 4 CF patients throughout the course of a pulmonary exacerbation. Patient 4 is the patient from which the isolates in the current study were collected. Patients 2, 3 and 4 were colonized with the PES strain while the Patient 1 had a non-PES strain. Patients 1 and 2 showed a decline in total *P. aeruginosa* levels and are defined as ‘responders’, i.e. the drop in cell count corresponded with administration of antibiotic therapy. Patients 3 and 4 did not show any drop in *P. aeruginosa* levels despite resolution of exacerbation symptoms and can defined as ‘non-responders’.
(PDF)

Table S1 **Sample Details.**
(PDF)
