# Supplementary material for: Phenotypic Heterogeneity of Pseudomonas aeruginosa Populations in a Cystic Fibrosis Patient
Source: PLoS One. 2013 Apr 3;8(4):e60225. doi: 10.1371/journal.pone.0060225 (PMC3616088; doi:10.1371/journal.pone.0060225)
Supplement: Figure S1 — Representative plate showing the diversity of colony morphologies of P. aeruginosa isolates from a single sputum sample. (PDF) [file pone.0060225.s001.pdf]

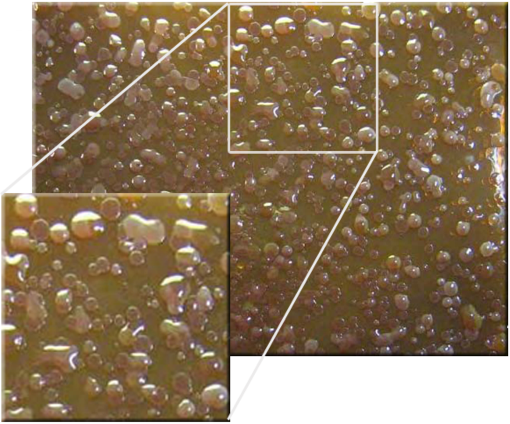

Figure S1. Representative plate showing the diversity of colony morphologies of *P. aeruginosa* isolates from a single sputum sample.
