# Supplementary material for: Phenotypic Heterogeneity of Pseudomonas aeruginosa Populations in a Cystic Fibrosis Patient
Source: PLoS One. 2013 Apr 3;8(4):e60225. doi: 10.1371/journal.pone.0060225 (PMC3616088; doi:10.1371/journal.pone.0060225)

Figure S2. Pulse-field gel electrophoresis. A UPGMA tree based on PFGE patterns across all the isolates compared using the Dice coefficient. The tree is identical to the one shown in Figure 2, but has been rooted to allow for display of the gel images.

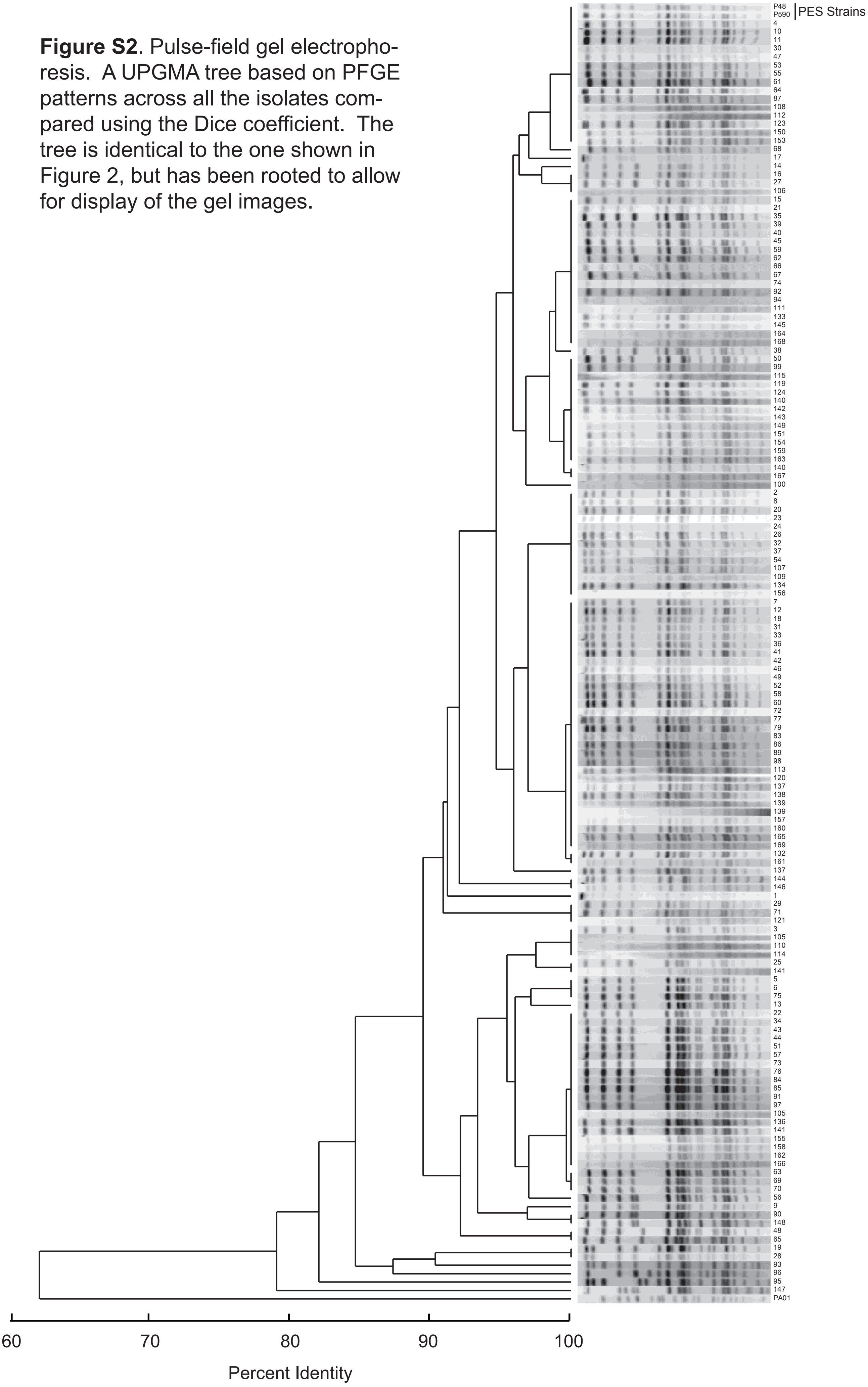

Supplement: Figure S2 — Pulse-field gel electrophoresis. A UPGMA tree based on PFGE patterns across all the isolates compared using the Dice coefficient. The tree is identical to the one shown in Figure 2, but has been rooted to allow for display of the gel images. (PDF) [file pone.0060225.s002.pdf]
