# Supplementary material for: Phenotypic Heterogeneity of Pseudomonas aeruginosa Populations in a Cystic Fibrosis Patient
Source: PLoS One. 2013 Apr 3;8(4):e60225. doi: 10.1371/journal.pone.0060225 (PMC3616088; doi:10.1371/journal.pone.0060225)
Supplement: Figure S3 — Variation in growth measures. Growth rates for each isolate under different media types are shown with error bars (standard deviation). Isolates were sorted according to their growth in M9 minimal media with glucose and case amino acids. (PDF) [file pone.0060225.s003.pdf]

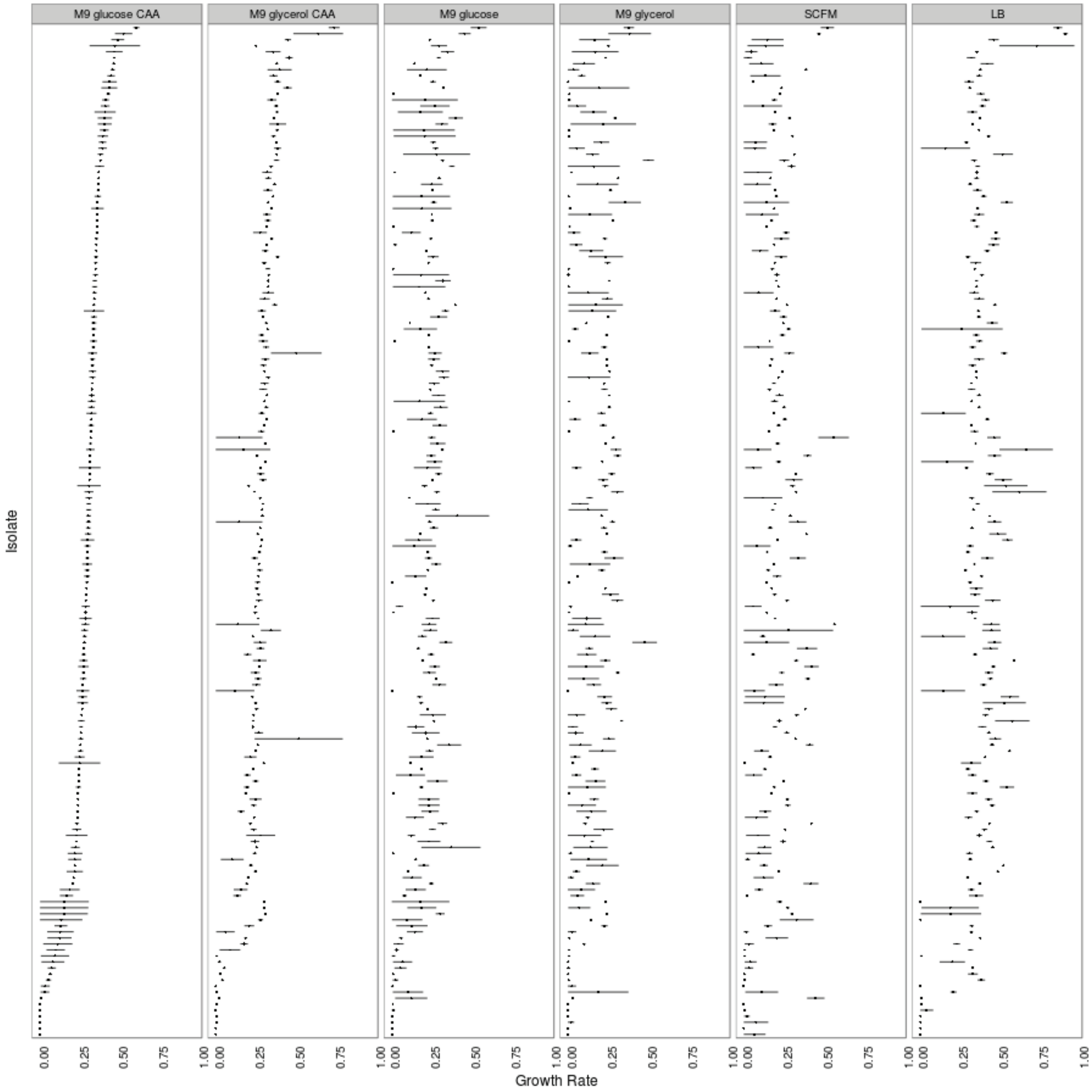

Figure S3. Variation in growth measures. Growth rates for each isolate under different media types are shown with error bars (standard deviation). Isolates were sorted according to their growth in M9 minimal media with glucose and case amino acids.
