# Supplementary material for: Phenotypic Heterogeneity of Pseudomonas aeruginosa Populations in a Cystic Fibrosis Patient
Source: PLoS One. 2013 Apr 3;8(4):e60225. doi: 10.1371/journal.pone.0060225 (PMC3616088; doi:10.1371/journal.pone.0060225)
Supplement: Figure S4 — Correlation matrix of the measured phenotypes. The Pearson correlation coefficient was calculated for each phenotype pair where the yellow represents a high correlation (close to 1) and blue represents no correlation (close to 0). (PDF) [file pone.0060225.s004.pdf]

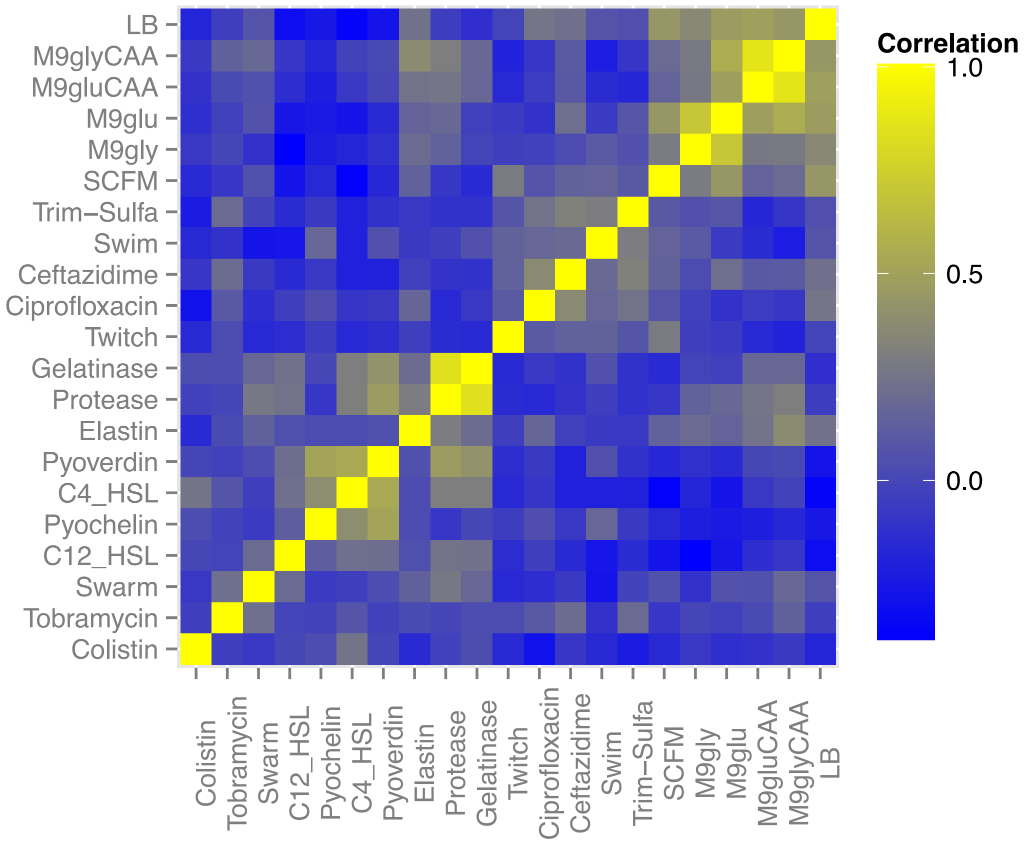

Figure S4. Correlation matrix of the measured phenotypes. The Pearson correlation coefficient was calculated for each phenotype pair where the yellow represents a high correlation (close to 1) and blue represents no correlation (close to 0).
