# Supplementary material for: Phenotypic Heterogeneity of Pseudomonas aeruginosa Populations in a Cystic Fibrosis Patient
Source: PLoS One. 2013 Apr 3;8(4):e60225. doi: 10.1371/journal.pone.0060225 (PMC3616088; doi:10.1371/journal.pone.0060225)
Supplement: Figure S5 — Changes in mean phenotype values over time. The mean of each phenotype was calculated for each sputum sample. The point are coloured according to the clinical status. A local smoothing function, shown with a blue line (shaded area represents 95% confidence intervals) was added to identify any trends present in the data. (PDF) [file pone.0060225.s005.pdf]

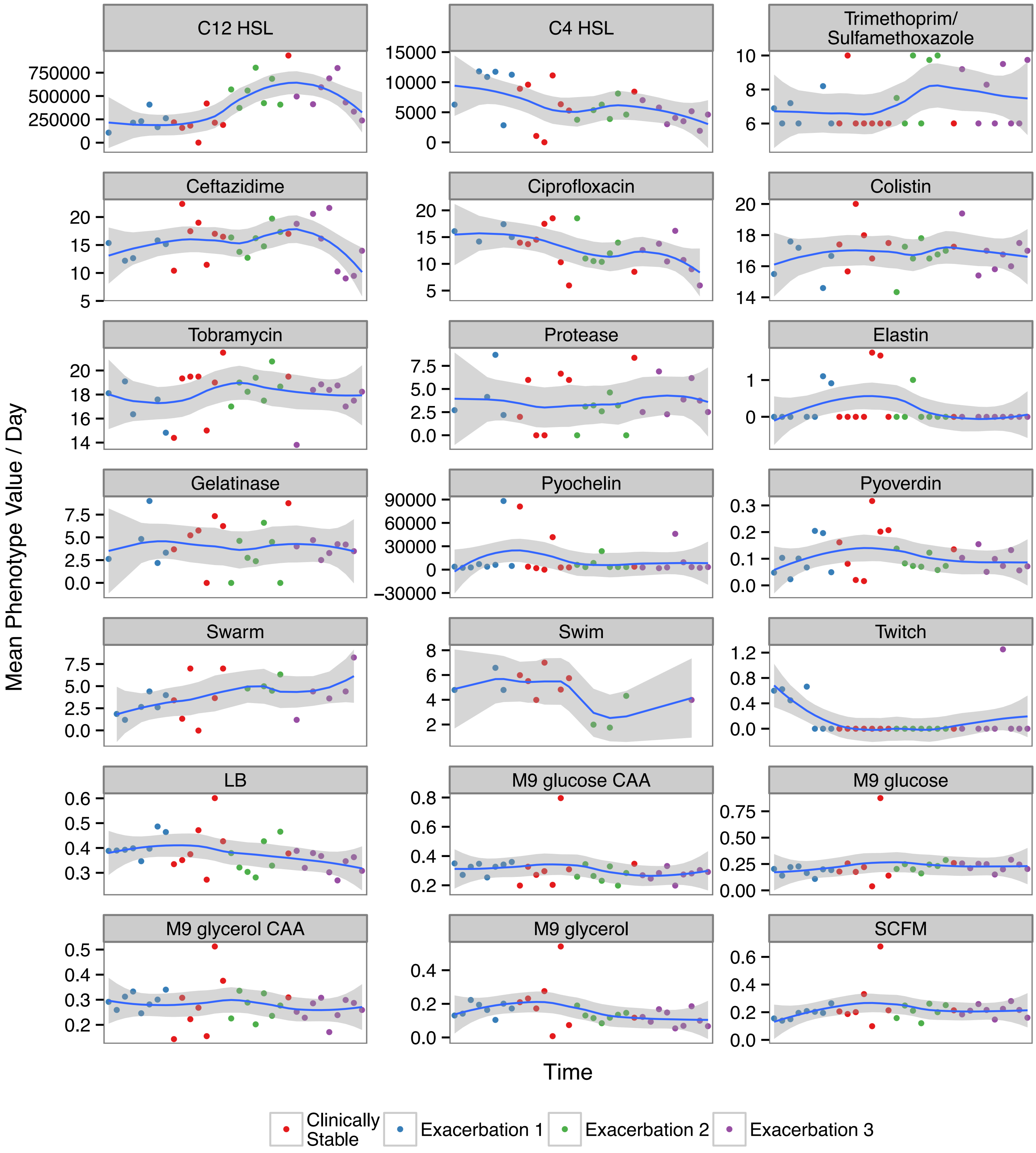

Figure S5. Changes in mean phenotype values over time. The mean of each phenotype was calculated for each sputum sample. The point are coloured according to the clinical status. A local smoothing function, shown with a blue line (shaded area represents 95% confidence intervals) was added to identify any trends present in the data.
