# Supplementary material for: Phenotypic Heterogeneity of Pseudomonas aeruginosa Populations in a Cystic Fibrosis Patient
Source: PLoS One. 2013 Apr 3;8(4):e60225. doi: 10.1371/journal.pone.0060225 (PMC3616088; doi:10.1371/journal.pone.0060225)
Supplement: Figure S6 — Antibiotic susceptibility profiles of the collected isolates during the three pulmonary exacerbations. Isolates were classified based on zone sizes from disk diffusion assays. For each sputum sample the relative proportion of resistant (red), intermediate (blue), and sensitive (green) isolates is shown for the four antibiotics where standardized cutoff values for P. aeruginosa are available. (PDF) [file pone.0060225.s006.pdf]

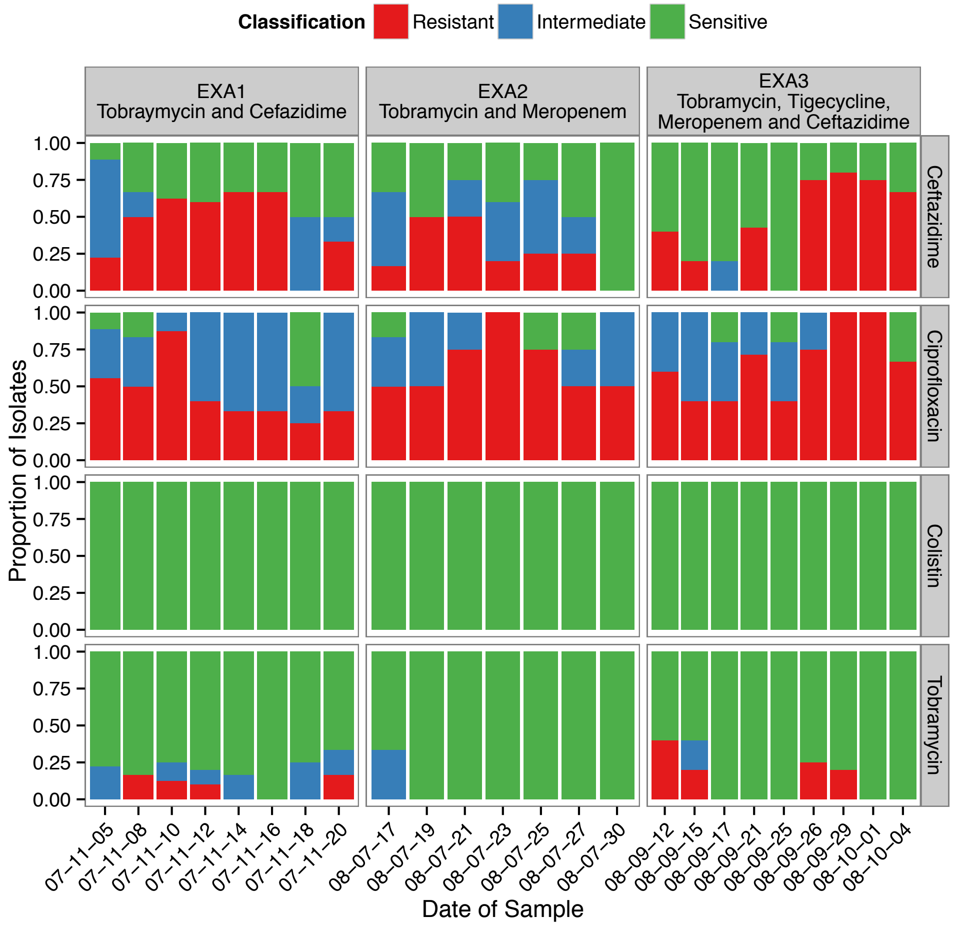

Figure S6. Antibiotic susceptibility profiles of the collected isolates during the three pulmonary exacerbations. Isolates were classified based on zone sizes from disk diffusion assays. For each sputum sample the relative proportion of resistant (red), intermediate (blue), and sensitive (green) isolates is shown for the four antibiotics where standarized cutoff values for *P. aeruginosa* are available.
