# Supplementary material for: Phenotypic Heterogeneity of Pseudomonas aeruginosa Populations in a Cystic Fibrosis Patient
Source: PLoS One. 2013 Apr 3;8(4):e60225. doi: 10.1371/journal.pone.0060225 (PMC3616088; doi:10.1371/journal.pone.0060225)
Supplement: Figure S7 — PCA analysis of the measured phenotype profiles. The first two components are shown and the data points are coloured according to the cluster pattern observed in the hierarchical cluster analysis. The amount of variation (in percent) explained by each principle component is shown on the axis. (PDF) [file pone.0060225.s007.pdf]

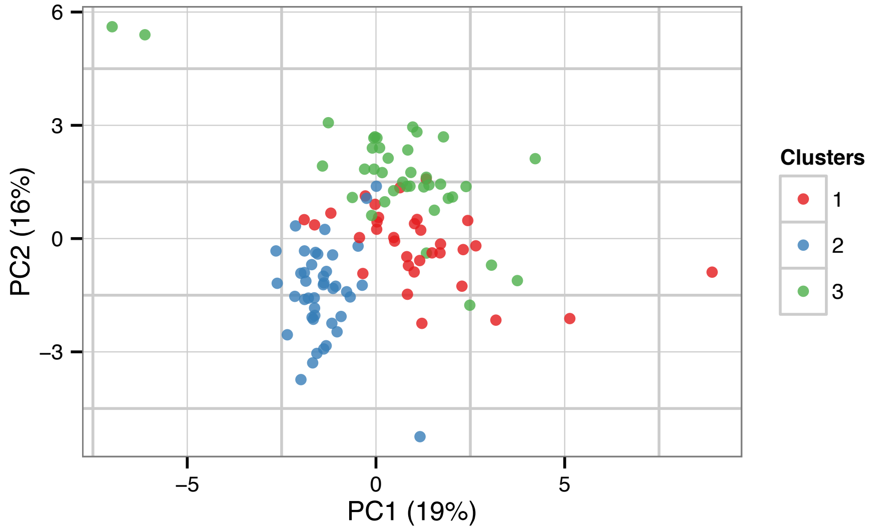

Figure S7. PCA analysis of the measure phenotype profiles. The first two components are shown and the data points are coloured according to the cluster pattern observed in the hierarchical cluster analysis. The amount of variation (in percent) explained by each principle component is shown on the axis.
