# Supplementary material for: Phenotypic Heterogeneity of Pseudomonas aeruginosa Populations in a Cystic Fibrosis Patient
Source: PLoS One. 2013 Apr 3;8(4):e60225. doi: 10.1371/journal.pone.0060225 (PMC3616088; doi:10.1371/journal.pone.0060225)
Supplement: Figure S8 — Phenotypic diversity of P. aeruginosa isolates from multiple CF patients. Isolates were collected from an additional 3 CF patients to determine the extent of phenotypic diversity. Samples were collected during a single exacerbation period and processed as before. Multiple isolates were collected from each sample, which often contained both mucoid (red) and non-mucoid (blue) isolates. Patient 1 was colonized with a non-PES strain while both patients 2 and 3 were colonized with PES. (PDF) [file pone.0060225.s008.pdf]

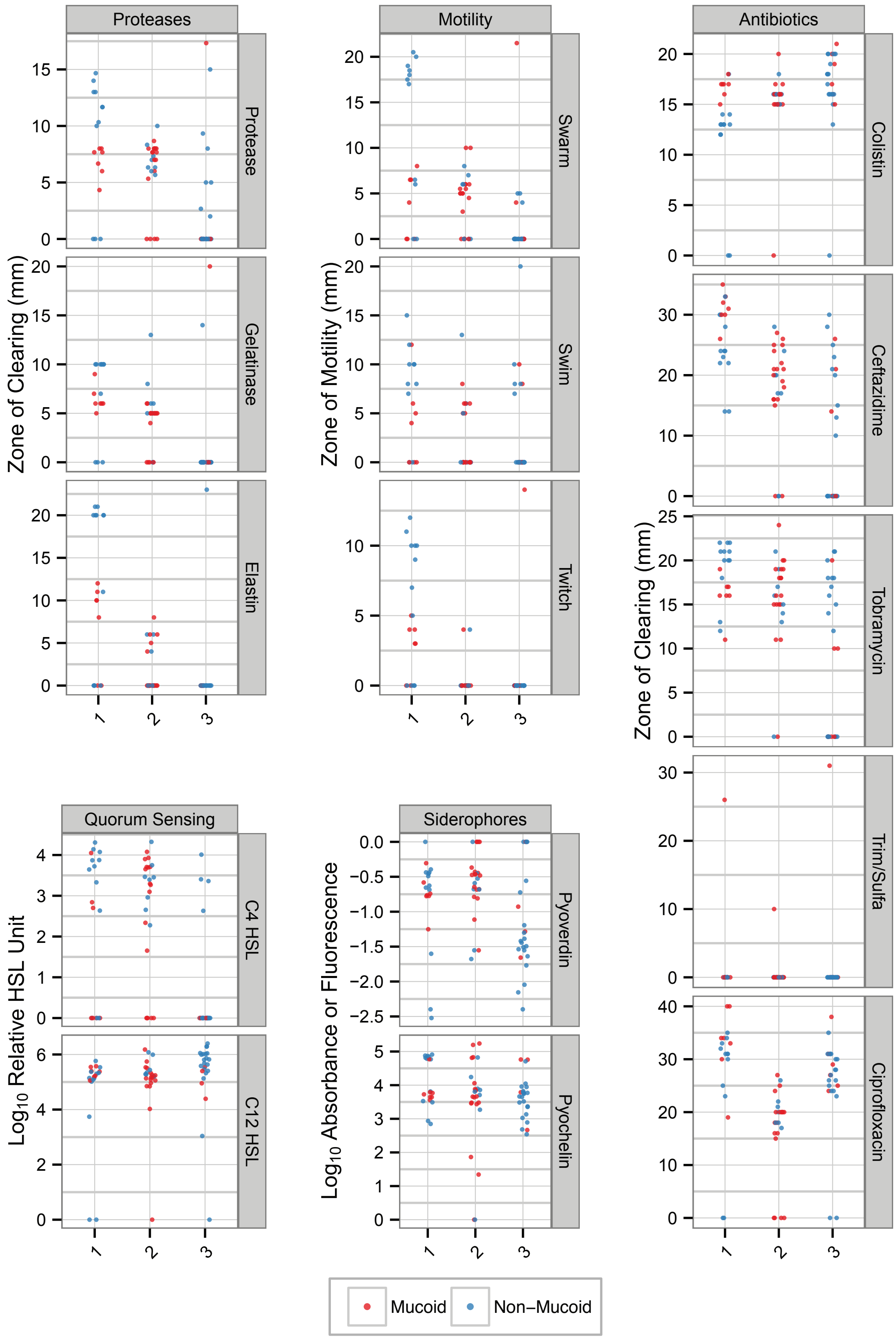

Figure S8. Phenotypic diversity of *P. aeruginosa* isolates from multiple CF patients. Isolates were collected from an additional 3 CF patients to determined the extent of phenotypic diversity. Samples were collected during a single exacerbation period and processed as before. Multiple isolates were collected from each sample, which often contained both mucoid (red) and non-mucoid (blue) isolates. Patient 1 was colonlize with a non-PES strain while both patients 2 and 3 were colonized with PES.
