# Supplementary material for: Phenotypic Heterogeneity of Pseudomonas aeruginosa Populations in a Cystic Fibrosis Patient
Source: PLoS One. 2013 Apr 3;8(4):e60225. doi: 10.1371/journal.pone.0060225 (PMC3616088; doi:10.1371/journal.pone.0060225)
Supplement: Figure S9 — Responders versus non-responders. Cell counts for mucoid (red), non-mucoid (blue), and total (green) P. aeruginosa in 4 CF patients throughout the course of a pulmonary exacerbation. Patient 4 is the patient from which the isolates in the current study were collected. Patients 2, 3 and 4 were colonized with the PES strain while the Patient 1 had a non-PES strain. Patients 1 and 2 showed a decline in total P. aeruginosa levels and are defined as ‘responders’, i.e. the drop in cell count corresponded with administration of antibiotic therapy. Patients 3 and 4 did not show any drop in P. aeruginosa levels despite resolution of exacerbation symptoms and can defined as ‘non-responders’. (PDF) [file pone.0060225.s009.pdf]

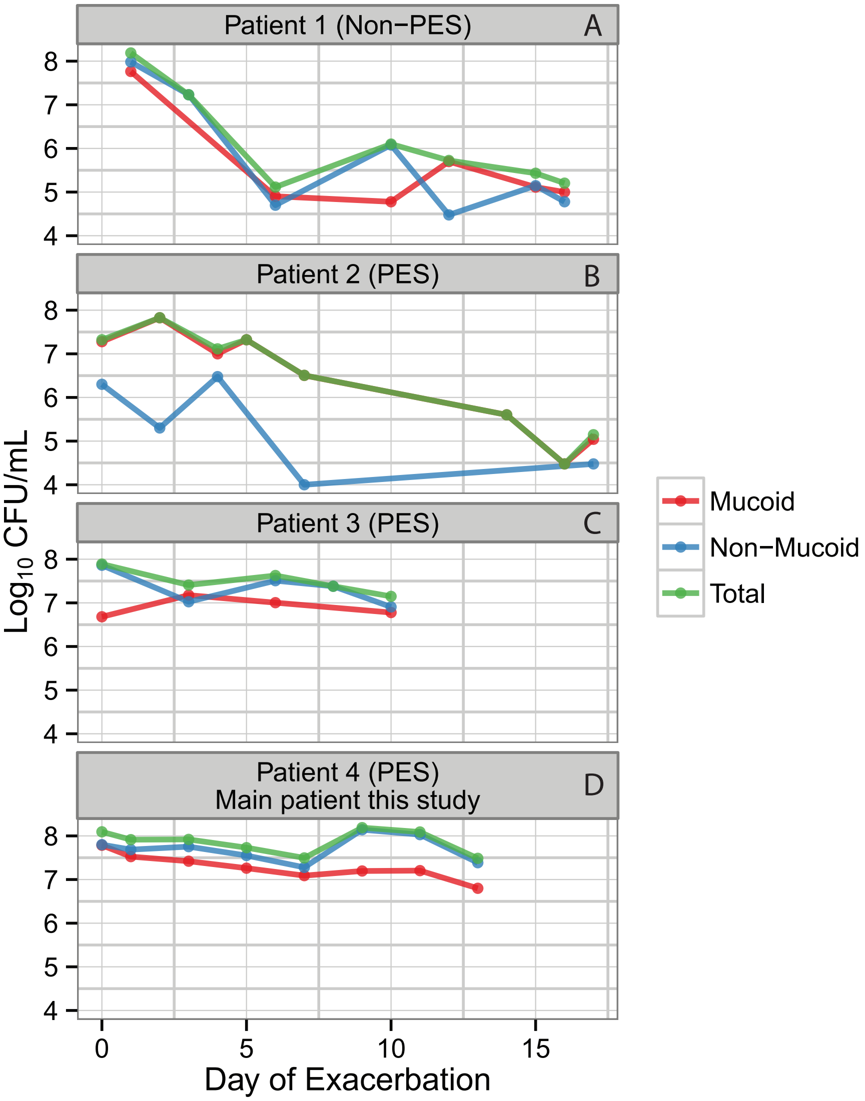

Figure S9. Responders versus non-responders. Cell counts for mucoid (red), non-mucoid (blue), and total (green) *P. aeruginosa* in 4 CF patients throughout the course of an pulmonary exacerbation. Patient 4 is the patient from which the isolates in the current study were collected. Patients 2, 3 and 4 were colonized with the PES strain while the Patient 1 had a non-PES strain. Patients 1 and 2 showed a decline in total *P. aeruginosa* levels and are defined as 'responders', i.e. the drop in cell count corresponded with administration of antibiotic therapy. Patients 3 and 4 did not show any drop in *P. aeruginosa* levels despite resolution of exacerbation symptoms and can defined as 'non-responders'.
