# Supplementary material for: Phenotypic Heterogeneity of Pseudomonas aeruginosa Populations in a Cystic Fibrosis Patient
Source: PLoS One. 2013 Apr 3;8(4):e60225. doi: 10.1371/journal.pone.0060225 (PMC3616088; doi:10.1371/journal.pone.0060225)
Supplement: Table S1 — Sample Details. (PDF) [file pone.0060225.s010.pdf]

Table S1. Sample details

Event	Date	Number of isolates	Antibiotic treatment during period
EXA1[a]	Nov 8-20, 2007	64	Parenteral tobramycin and ceftazidime
CS1[b]	Feb 4, 2008	3	Inhaled tobramycin alternating with oral ciprofloxacin each sequentially for 2 weeks on/off
CS2	April 16, 2008	2	Inhaled tobramycin alternating with oral ciprofloxacin each sequentially for 2 weeks on/off
CS3	May 1, 2008	5	Inhaled tobramycin alternating with oral ciprofloxacin each sequentially for 2 weeks on/off
CS4	May 8, 2008	5	Inhaled tobramycin alternating with oral ciprofloxacin each sequentially for 2 weeks on/off
CS5	May 12, 2008	5	Inhaled tobramycin alternating with oral ciprofloxacin each sequentially for 2 weeks on/off
CS6	June 16, 2000	6	Inhaled tobramycin alternating with oral ciprofloxacin each sequentially for 2 weeks on/off
EXA2	July 17-30, 2000	30	Parenteral tobramycin and meropenem
CS7	Aug 12, 2008	4	Inhaled tobramycin alternating with inhaled colistin each sequentially for 2 weeks on/off
EXA3	Sept 17- Oct 4, 2008	45	Parenteral tobramycin, tigecycline, meropenem, and ceftazidime

[a]EXA, exacerbation period (hospitalization required)
[b]CS, clinically stable period (routine clinic visit)
